# The combination of HDAC and aminopeptidase inhibitors is highly synergistic in myeloma and leads to disruption of the NFκB signalling pathway

**DOI:** 10.18632/oncotarget.1168

**Published:** 2013-08-12

**Authors:** Emma M. Smith, Lei Zhang, Brian A. Walker, Emma L. Davenport, Lauren I. Aronson, David Krige, Leon Hooftman, Alan H. Drummond, Gareth J. Morgan, Faith E. Davies

**Affiliations:** ^1^ Haemato-Oncology Research Unit, Division of Molecular Pathology, Cancer Therapeutics and Clinical Studies, The Institute of Cancer Research, London, UK; ^2^ Chroma Therapeutics Ltd., Abingdon, UK

**Keywords:** myeloma, HDAC inhibitor, aminopeptidase inhibitor, NFκB

## Abstract

There is a growing body of evidence supporting the use of epigenetic therapies in the treatment of multiple myeloma. We show the novel HDAC inhibitor CHR-3996 induces apoptosis in myeloma cells at concentrations in the nanomolar range and with apoptosis mediated by p53 and caspase pathways. In addition, HDAC inhibitors are highly synergistic, both *in vitro* and *in vivo*, with the aminopeptidase inhibitor tosedostat (CHR-2797). We demonstrate that the basis for this synergy is a consequence of changes in the levels of NFκB regulators BIRC3/cIAP2, A20, CYLD, and IκB, which were markedly affected by the combination. When co-administered the HDAC and aminopeptidase inhibitors caused rapid nuclear translocation of NFκB family members p65 and p52, following activation of both canonical and non-canonical NFκB signalling pathways. The subsequent up-regulation of inhibitors of NFκB activation (most significantly BIRC3/cIAP2) turned off the cytoprotective effects of the NFκB signalling response in a negative feedback loop. These results provide a rationale for combining HDAC and aminopeptidase inhibitors clinically for the treatment of myeloma patients and support the disruption of the NFκB signalling pathway as a therapeutic strategy.

## INTRODUCTION

Despite the recent introduction of novel therapies for the treatment of the plasma cell malignancy multiple myeloma, it remains an almost incurable disease with a high rate of relapse. At relapse the clonal cells are often resistant to standard therapies and there is a clinical need to develop novel therapeutic combinations. Epigenetic processes such as DNA methylation and regulation of chromatin structure are now considered to make an important contribution to gene regulation, oncogenic transformation and survival of neoplastic cells in a wide range of malignancies including myeloma [1, 2]. These epigenetic changes can be targeted therapeutically and there is a growing body of early data supporting the clinical efficacy of epigenetic therapies in myeloma [2].

Histone deacetylases (HDACs) are one of the best characterised components of the epigenetic machinery and encompasses a family of 18 members [3]. A major function of HDACs is to catalyse the removal of acetyl groups from histone tails which, in concert with other epigenetic mechanisms, promotes the condensation of DNA into transcriptionally inactive heterochromatin [4]. In addition to targeting histones, the de-acetylating activity of HDACs also affects numerous non-histone targets. These acetylated proteins or the ‘acetylome’ include transcription factors p53 [5, 6], STAT3 [7, 8], and the NFκB family member p65 [9-11], a target that may be of particular relevance in myeloma cells which are recognised as being highly dependent on NFκB signalling [12]. Furthermore, HDAC6 has been shown to regulate the processing of misfolded proteins via the aggresome pathway [13, 14], a process that is particularly important to myeloma cells which have to cope with a significant load of unfolded immunoglobulin[15, 16]. These diverse roles of HDACs imply that cell death resulting from their inhibition is likely to be multi-factorial and depend upon the selective activity of the inhibitor used against the different HDAC family members.

Recently developed HDAC inhibitors, such as vorinostat, panobinostat, and romidepsin have shown some promising anti-tumour activities in multiple myeloma but only modest effects in the clinical setting [17-19]. Combination studies in a number of clinical trials also support the use of HDAC inhibitors with other anti-tumour agents such as bortezomib [20], but dose limiting toxicities are frequently observed and efficacy needs to be improved. A better understanding of the mechanisms of HDAC inhibitors is required to overcome the current challenges in single and combination strategies, while new HDAC inhibitors and novel combination strategies may result in toxicity reduction and better patient outcomes.

In this study we describe the effects of a novel HDAC inhibitor, CHR-3996, on myeloma cells. CHR-3996 demonstrates HDAC inhibitory activity at low concentrations with minimal activity against HDAC6 function and the aggresome pathway [21]. We show that this HDAC inhibitor is highly efficacious against myeloma cells, inducing apoptosis via its effects on the acetylome, and go on to demonstrate that it is highly synergistic when combined with the aminopeptidase inhibitor tosedostat (CHR-2797) [22], which we have previously shown to have potent anti-myeloma activity *in vitro* and *in vivo* [23, 24]. Aminopeptidase inhibitors act downstream of the proteasome, catalysing the breakdown of proteasome generated peptides into their constituent amino acids. However, unlike proteasome inhibitors, the inhibition of aminopeptidases does not result in the toxic build-up of misfolded/aggregated proteins, so they have a different biological impact to proteasome inhibitors. Inhibiting aminopeptidases has been shown to starve the cell of essential amino acids and trigger the amino acid deprivation response [22, 23]. The combination of these compounds leads to rapid activation of NFκB signalling and a subsequent induction of a negative feedback loop, mediated via NFκB regulators such as BIRC3, resulting in myeloma cell death.

## RESULTS

### HDAC inhibition induces apoptosis of myeloma cells

**Figure 1:**
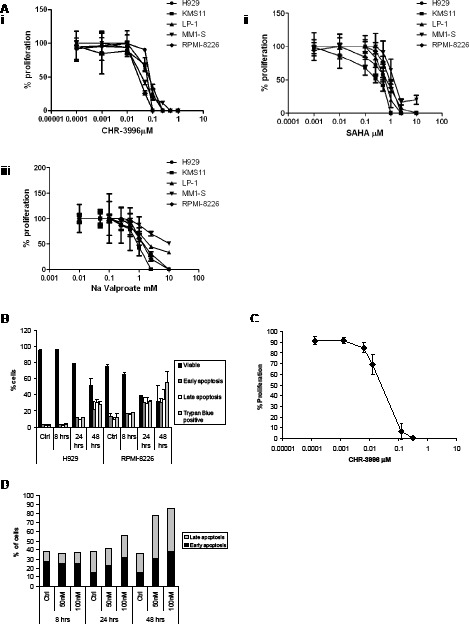
CHR-3996 inhibits the proliferation of myeloma cells and induces apoptosis **A.** A panel of myeloma cell lines were treated with a range of concentrations of CHR-3996 (i), SAHA (ii), or Sodium (Na) Valproate (iii) for 48 hours. The proliferation of the cells was monitored by metabolic activity (WST-1 assay) and is shown as a percentage of untreated cells. The LC_50_ for each cell line is shown in Supplementary Table 1. **B.** H929 and RPMI-8226 cells were treated with CHR-3996 (250 nM and 100 nM respectively) for 8, 24, or 48 hours. Apoptosis was analysed by Annexin-FITC/propidium iodide (PI) staining and analysis by flow cytometry (AnnexinV positive is defined as early apoptotic, AnnexinV and PI positive as late apoptotic) and by trypan blue exclusion. **C.** Primary CD138^+^ plasma cells from three patients were treated with a range of concentrations of CHR-3996 and the proliferation measured by WST-1 assay. **D.** Primary patient CD138^+^ plasma cells were treated with either 50 or 100 nM CHR-3996 over a time-course of 48 hours. The percentage of cells undergoing apoptosis was determined by binding of AnnexinV and PI followed by analysis by flow cytometry. Results from a representative patient are shown.

CHR-3996 was shown to inhibit the proliferation of a panel of myeloma cells using the WST-1 assay based on metabolic activity (Figure 1A). The LC_50_ values for the various cell lines treated with CHR-3996 ranged from 30.3-97.6nM. In comparison to two commercially available HDAC inhibitors, SAHA and Sodium Valproate, CHR-3996 was shown to be effective at much lower concentrations and was approximately 10-fold more potent than SAHA and 10-thousand fold more potent than Sodium Valproate (Supplementary Table 1). For further experiments in two cell lines with different translocations, H929 t(4;14) and RPMI-8226 t(16;22), both associated in patients with poor prognoses and needing alternate therapeutic strategies, were selected and treated with CHR-3996. To ascertain whether CHR-3996 is cytostatic or cytotoxic, these cell lines were treated with CHR-3996 and the percentage of cells undergoing apoptosis was determined (Figure 1B). The percentage of viable cells decreased to around 50% in both cell lines and there was a concomitant increase in early and late apoptotic cells indicating that CHR-3996 induces cell death. CD138^+^ plasma cells isolated from myeloma patients were also sensitive in a WST-1 assay to CHR-3996 treatment at doses comparable to MM cell lines (average=18nM) (Figure 1C). CHR-3996 also induced apoptosis in primary patient cells in a dose-dependent manner: 24 hours following treatment there was an increase in the percentage of cells in early apoptosis and at 48 hours there was more than a two-fold increase in the percentage of both early and late apoptotic cells compared to untreated cells (Figure 1D).

### CHR-3996 induces apoptosis via p53-dependent pathways and caspase activation

**Figure 2:**
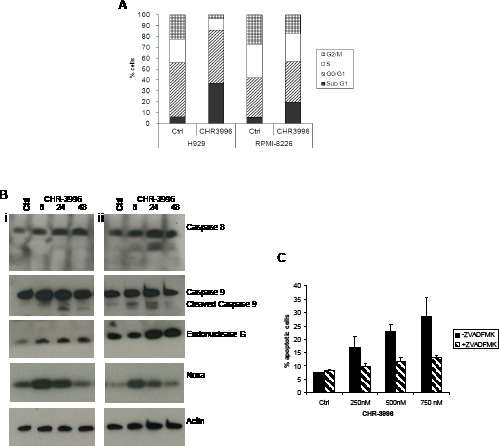
CHR-3996 induces cell cycle arrest and apoptosis via caspase-dependent and independent pathways **A.** H929 or RPMI-8226 cells were treated with CHR-3996 at 250 or 100 nM respectively for 48 hours, following which the cells were fixed in alcohol, treated with RNase, then stained with Propidium Iodide (PI) and analysed by flow cytometry. **B.** H929 (i) and RPMI-8226 (ii) cells were treated with CHR-3996 at 250 or 100 nM respectively and cells lysed for protein at 8, 24, and 48 hours and used for immunoblotting. **C.** H929 cells were pre-treated with Z-VAD-FMK (50 μM) for 1.5 hours prior to addition of varying concentrations of CHR-3996 (250 nM). The cells were harvested and stained with annexinV and PI and analysed by flow cytometry. Cells staining positive for annexinV alone or annexinV and PI were defined as apoptotic. A representative of three experiments is shown.

HDAC inhibitors have been widely reported to arrest cell cycle progression and up-regulate cyclin dependent kinase (cdk) inhibitors such as CDKN1A (p21) expression [25]. To test whether CHR-3996 has similar effects in myeloma cells, H929 and RPMI-8226 cells were treated with CHR-3996 and PI binding was used to determine the cellular DNA content (Figure 2A). There was evidence of a G0/G1 block in cell cycle progression; the percentage of H929 cells in G1 remained at 48%, whilst the percentage of cells in S and G2/M phase decreased from 21 to 10% and 22 to 3.5% respectively. Likewise for RPMI-8226 cells the percentage of cells in S phase fell from 30 to 25% and G2/M from 26 to 16%. Additionally, both myeloma cell lines demonstrated an increase in the percentage of cells in the subG0/G1 fraction (H929 from 5.8 to 35.6%, RPMI-8226 from 5.3 to 18.7%), indicating that drug treatment induced apoptosis.

Apoptosis in response to CHR-3996 was shown to occur mainly via caspase-dependent mechanisms (Figure 2B). The pro-apoptotic DNase EndonucleaseG was up-regulated, as was the p53 down-stream mediator Noxa (Figure 2B). Treatment of H929 and RPMI-8226 cells led to the cleavage of caspase 9 with minimal involvement of the extrinsic apoptosis mediator caspase 8. The demonstration of caspase 9 cleavage indicates that activation of the proteolytic pathway is a hallmark of CHR-3996 induced apoptosis. To further clarify the role of caspases in cell death, H929 cells were pre-treated with the pan-caspase inhibitor Z-VAD-FMK 1.5 hours prior to exposure to CHR-3996. Following 24 hours there was a dose-dependent increase in the percentage of cells undergoing apoptosis in the presence of CHR-3996; however this was largely inhibited by pre-treatment with Z-VAD-FMK (Figure 2C). At the highest concentration of CHR-3996 (750 nM) 35% of cells were apoptotic whereas this was reduced to 13.9% following addition of Z-VAD-FMK, only slightly higher than baseline levels of apoptosis without CHR-3996 treatment (8.5%).

### The effects of CHR-3996 on the bone marrow microenvironment

**Figure 3:**
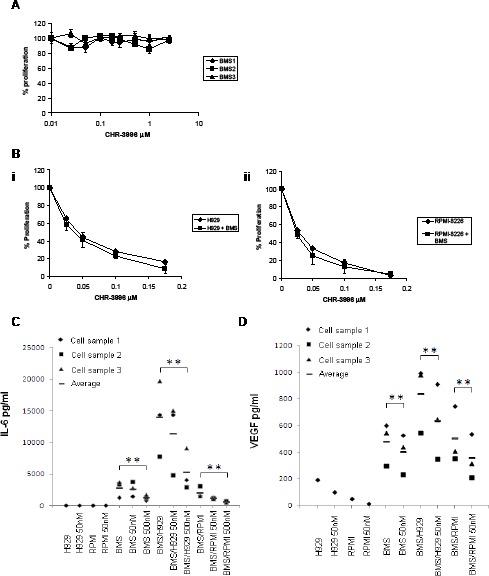
CHR-3996 overcomes the protective effect of the bone marrow microenvironment Bone marrow stromal cells were cultured from bone marrow aspirates obtained from three patients with myeloma. **A.** The bone marrow stromal cells were treated with a range of CHR-3996 concentrations for 24 hours. The number of viable cells was measured by metabolic activity (WST-1 assay) and is shown as a percentage of untreated cells. **B.** H929 (i) or RPMI-8226 (ii) cells were treated with CHR-3996 in the presence or absence of the bone marrow stromal cells (performed in triplicate) for 24 hours. The proliferation of the cells was monitored by metabolic activity (WST-1 assay) and is shown as a percentage of untreated cells. IL-6 **C.** and VEGF **D.** secreted by the bone marrow stromal cells over 24 hours in isolation or in a co-culture with H929 or RMPI-8226 cells and with or without CHR-3996 treatment were measured by ELISA. The data was compared using a one-tailed paired Student's T Test and ** indicates a p value of <0.05.

To test whether CHR-3996 can overcome survival factors provided by the bone marrow microenvironment, myeloma cell lines were grown in the presence of bone marrow stromal cells. The bone marrow stromal cells themselves did not show altered proliferation in response to a range of CHR-3996 concentrations up to 2.5 μM (Figure 3A), but the myeloma cell lines H929 and RPMI-8226 remained highly sensitive to CHR-3996 showing no difference in cell viability across a range of doses in the presence or absence of bone marrow stromal cells (Figure 3B). Although CHR-3996 did not induce apoptosis in bone marrow stromal cells, there was a significant reduction in the levels of IL-6 and VEGF they secreted (Figure 3C-3D) that decreased in a dose-dependent manner. Since IL-6 and VEGF are secreted by both myeloma and stromal cells, we studied the level of IL-6 and VEGF in co-culture media. In untreated co-culture conditions, the variations observed in cytokine secretion between co-culture samples reflect the varied ability of different stroma cell samples to induce cytokine production from MM cell lines. This observation supports the hypothesis that myeloma survival is largely dependent on the bone marrow microenvironment. Similar reductions in levels of both cytokines were observed in the culture media when bone marrow stromal cells were co-cultured with myeloma cell lines H929 and RPMI-8226 (Figure 3C-3D), confirming CHR-3996 can modify the bone marrow microenvironment to adversely affect myeloma cell survival.

### CHR-3996 inhibits histone deacetylation in myeloma cells without affecting aggresome or proteasome function

**Figure 4:**
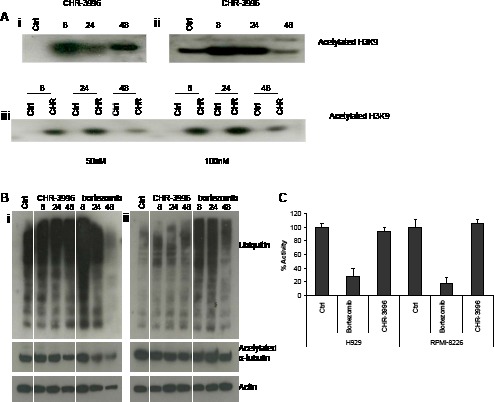
CHR-3996 treatment increases levels of acetylated histone H3K9 but does not affect levels of ubiquitinated proteins, acetylated alpha-tubulin, or inhibit proteasome function **A.** H929 (i) and RPMI-8226 (ii) cells were treated with CHR-3996 (250 and 100 nM respectively) and primary patient CD138^+^ plasma cells (iii) were treated with either 50 (left panel) or 100 nM (right panel) CHR-3996 over a time-course of 48 hours. Following cell lysis histones were released from DNA by overnight extraction with 0.2M HCl and immunoblotting performed to detect acetylated H3K9. **B.** H929 (i) and RPMI-8226 (ii) cells were treated with CHR-3996 (250 and 100 nM respectively) or bortezomib (8nM) for 8, 24, and 48 hours. Following cell lysis immunoblotting was performed to detect ubiquitin, acetylated a-tubulin, and actin. Crtl represents untreated cells. **C.** H929 and RPMI-8226 cells were treated with CHR-3996 (250 and 100 nM respectively) or bortezomib (4 nM). Following cell lysis 25 μg of protein was added to Suc-Leu-Leu-Val-Tyr-AMC, substrate for the chymotryptic activity of the proteasome, and the fluoresence read from each well every 120 seconds for 48 repeats. The activity was calculated from the rate of fluorescence detected in the linear phase of the reaction and shown as a percentage of untreated cells.

HDACs are known to contribute to the epigenetic control of gene expression by deacetylating histones promoting condensation of DNA into heterochromatin and repressing transcriptional activity in these regions. The novel compound CHR-3996 is shown to effectively inhibit histone-specific HDAC activity demonstrated by the increase of acetylated H3K9 in myeloma cell lines and also in primary patient myeloma cells in a time and dose-dependent manner (Figure 4A). Increases in acetylated H3K9 were observed early (8 hours) after exposure to CHR-3996 and high levels were maintained over 48 hours. Previous reports have demonstrated that specific inhibition of HDAC6 leads to the accumulation of acetylated α-tubulin [26, 27] and ubiquitinated proteins following disruption to aggresomes [13]. There was no determinable effect of CHR-3996 on the levels of acetylated α-tubulin or ubiquitinated proteins in myeloma cell lines H929 and RPMI-8226 (Figure 4B), indicating low activity of this compound against HDAC6. There was also no effect on the chymotryptic proteasome activity (Figure 4C). In contrast, Bortezomib, a well-characterised proteasome inhibitor, effectively inhibited proteasome activity by around 80% and concomitantly increased the levels of ubiquitinated cellular protein in both myeloma cell lines (Figure 4B-4C).

### Gene expression profiling of myeloma cells treated with HDAC inhibitor

In order to determine the cellular changes associated with the onset of cell cycle arrest and apoptosis, we performed gene expression profiling on H929 cells that had been exposed to 250 nM CHR-3996 (NCBI's Gene Expression Omnibus [28] GEO Series accession number GSE20405 (http://www.ncbi.nlm.nih.gov/geo/query/acc.cgi?acc=GSE20405) (Supplementary Table 2).

Treating the cells with the HDAC inhibitor affected the expression of numerous genes, a large proportion of which were involved in the regulation of cell cycle. Inhibitors of cyclin dependent kinases and genes mediating DNA damage-induced cell cycle arrest increased in expression, while genes stimulating initiation of mitosis and progression of cell cycle decreased. There were also changes observed in the level of expression of key regulators of the NFκB signalling pathway *BIRC3*, *CYLD*, *TRAF1* and *BCMA*. The expression changes seen support the presence of increased p53 signalling, as evidenced by alterations in expression of p53 regulated genes, in addition to which negative inhibitors of p53, such as *MDM2*, decreased following HDAC inhibitor treatment. Increases in the expression of key mediators of stress signalling, such as *PERK*, *CHOP* and *IRE-1* indicate the cells experienced higher levels of endoplasmic reticulum mediated cellular stress following HDAC inhibition. The induction of apoptosis was reflected in increases in the expression levels of the pro-apoptotic proteins *BIM*, *PUMA, FOXO3* and *APAF1*, together with a decrease in the anti-apoptotic protein *BIRC5* (*survivin*). CHR-3996 appears to down-regulate the pro-survival Wnt signalling pathway because inhibitors of Wnt signalling were more highly expressed after exposure to this compound. There is also evidence that the process of autophagy was up-regulated as indicated by the increased expression of *LC3B*, *APG5* and *-10*. Interestingly, there were also changes seen in the expression of chromatin modifying enzymes including increased *HDAC5* and *-9*, possibly a reflection of the cells trying to overcome the deleterious effects of the HDAC inhibitor.

### CHR-3996 is synergistic with an aminopeptidase inhibitor *in vitro*

Building effective clinical combinations *in vitro* is an important aim of translational drug development and in order to investigate whether CHR-3996 is more effective as part of a combination therapy, H929 and RPMI-8226 cells were treated with CHR-3996 together with either melphalan or bortezomib. A full Chou-Talalay analysis with 16 points on the curve revealed that while there was low level synergy with melphalan (a CI of around 0.7 in both cell lines), the combination of CHR-3996 and bortezomib was not synergistic (a CI of 1.3 for both cell lines). In a search for an agent giving rise to high level synergy we went on to examine the combination with an aminopeptidase inhibitor, tosedostat (CHR-2797), which we have recently shown to have potent anti-myeloma activity *in vitro* and *in vivo* [23, 24]. When CHR-3996 or two other widely studied HDAC inhibitors (SAHA and Sodium Valproate) were added to cell lines concomitantly with tosedostat the agents were highly synergistic (<0.7); an effect that became more profound when the aminopeptidase inhibitor was added to the cells 24 hours prior to the HDAC inhibitor (>0.4) (Supplementary Table 3). However, if the HDAC inhibitor was added prior to the tosedostat a smaller degree of synergy was observed and in some cases became antagonistic, suggesting the aminopeptidase inhibitor is sensitizing the cells to the effects of the HDAC inhibitor.

### CHR-3996 is synergistic with an aminopeptidase inhibitor *in vivo*

**Figure 5:**
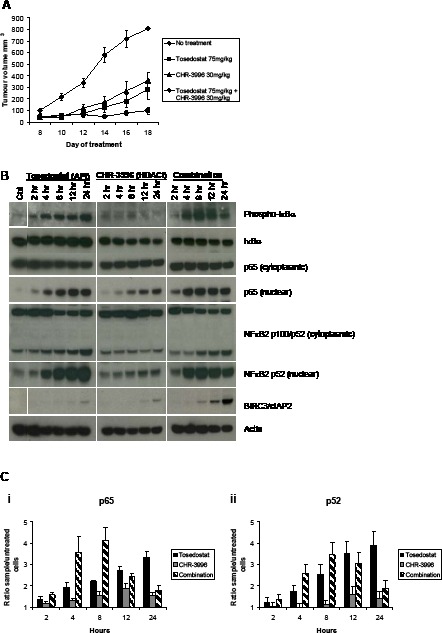
CHR-3996 is synergistic with Tosedostat (CHR-2797) in an in vivo model and causes rapid activation followed by down-regulation of NFκB signalling **A.** NOD/SCID IL2R^−/−^ mice were inoculated subcutaneously with 2×10^6^ H929 cells. Four days following the inoculation mice were administered CHR-3996, tosedostat, or a combination of the two agents on a daily basis. The tumours were measured every other day with callipers. The median tumour volume (calculated by 1/2(length)(width)^2^) is shown on the graph, Each treatment group n=10. **B.** and **C.** H929 cells were treated with tosedostat (CHR-2797) (APi) 1 μM, CHR-3996 (HDACi) 250 nM, or both compounds simultaneously and cytoplasmic/nuclear protein extracts prepared at the times indicated in the figure for **B.** immunoblotting and **C.** an NFκB DNA binding assay for p65 (i) and p52 (ii).

Administration of CHR-3996 in a NOD/SCID IL2R gamma^null^ xenograft model effectively inhibited tumour growth at doses that were well tolerated (Figure 5A). To interrogate whether the HDAC inhibitor and aminopeptidase inhibitor were synergistic as observed *in vitro*, doses of each agent were chosen that partially inhibited tumour growth (CHR-3996 30 mg/kg, tosedostat 75 mg/kg). These compounds were co-administered over a maximum of 28 days. Each agent in isolation slowed tumour growth but completely blocked tumour growth when the aminopeptidase inhibitor was added prior to the HDAC inhibitor (Figure 5A). Although *in vitro* studies showed a more profound effect, sequential dosing was not studied *in vivo* due to the pharmacokinetic properties of the two compounds meaning daily administration of both compounds was required, making sequential dosing difficult.

### The combination of CHR-3996 with an aminopeptidase inhibitor affects NFκB signalling

In order to understand the basis of the synergy we have demonstrated, H929 cells were treated with CHR-3996, tosedostat, or a combination of the two for 24 hours, then RNA extracted for global gene expression analysis. The largest fold changes in expression observed were those of negative NFκB regulators BIRC3, IκBα, A20, and CYLD (Table 1), suggesting that the combination may effectively target the NFκB pathway. To further investigate the effects of the HDAC and aminopeptidase inhibitors on NFκB signalling, immunoblotting was performed to analyse the phosphorylation status and localisation of key proteins in this pathway. Exposure of H929 cells to tosedostat alone and in combination with the HDAC inhibitor led to the activation of the canonical NFκB signalling pathway, demonstrated by increased IκBα phosphorylation and p65 translocation to the nucleus, and the non-canonical NFκB signalling pathway, shown by the proteolytic cleavage of p100 into the active form p52 (and its subsequent nuclear translocation) (Figure 5B). Treatment with CHR-3996 in isolation led to a modest increase in nuclear p65 (associated with decreased cytoplasmic levels of IκBα rather than IκBα phosphorylation) and a small increase in nuclear p52 detected, suggesting that the HDAC inhibitor alone has minimal impact on canonical or non-canonical NFκB activation (Figure 5B). Comparing tosedostat alone with the combination, the presence of the HDAC inhibitor led to a more rapid phosphorylation of IκB and nuclear translocation of p65 and p52, consistent with the faster activation of canonical and non-canonical NFκB pathways. However, by 12 hours there was a significant up-regulation of BIRC3/cIAP2 – an effect not seen with either compound alone – which was associated with subsequent decreases in IκBα phosphorylation, p65 and p52 nuclear translocation (Figure 5B). To further confirm these data, a DNA binding assay was performed to determine whether p65 and p52 detected in the nuclear fraction represented active transcription factors. Verifying the immunoblotting results, the HDAC inhibitor alone had a very small effect of levels of nuclear p65 and p52, whilst exposure to the aminopeptidase inhibitor increased the amount of p65 (Figure 5Ci) and p52 (Figure 5Cii) able to bind the NFκB consensus oligonucleotide sequence over 24 hours. When combined with the HDAC inhibitor the amount of active p65 and p52 in the nucleus increased more rapidly than following tosedostat treatment alone, but declined following 8 hours of treatment, confirming the results seen by immunoblotting.

**Table 1:** GEP changes following HDAC inhibition in myeloma cells

Probe ID	Gene	Fold change		
		APi	HDACi	Combination
210538_s_at	BIRC3 3	49.17	23.51	110.62
202644_s_at	A20	14.63	NS	16.64
201502_s_at	IκBα	4.93	NS	6.55
221903_s_at	CYLD	2.04	2.47	4.16

H929 cells were treated with tosedostat (CHR-2797) 1 μM (APi), CHR-3996 250 nM (HDACi), or a combination of both for 24 hours then analysed on a U133 Plus 2.0 Array (Affymetrix) and a supervised analysis performed with dCHIP comparing the groups of treated cells to untreated cells. All p values were less than 0.05. NS, no significant change.

## DISCUSSION

In this study we have shown that the HDAC inhibitor, CHR-3996, has anti-myeloma activity at low concentrations, leading to cell cycle arrest and apoptosis in myeloma cell lines and primary patients cells. CHR-3996 induced apoptosis was shown to be largely dependent on caspase activation and overcame the protective effects of bone marrow stromal cells. Whilst no loss of bone marrow stromal cell viability was observed, the amount of pro-myeloma cytokines IL-6 and VEGF secreted decreased significantly. Numerous other HDAC inhibitors have been shown to have anti-myeloma activities, including Sodium Valproate [29], Sodium Butyrate [25], Trichostatin A (TSA) [25], SAHA [30], FR901228 (depsipeptide) [31], LBH589 [32], PXD101 [33], ITF2357 [34], KD5170 [35], tubacin [14], and NVP-LAQ824 [36]. Many of these data are in agreement with our results, showing that HDAC inhibitors induce apoptosis in myeloma cells, however, the mechanism for this remains controversial. The HDAC inhibitors LBH589, NVP-LAQ824, and KD5170 demonstrated similar findings to CHR-3996 and were found to induce cell death via activation of the caspase pathway with the pan-caspase inhibitor protecting the myeloma cell lines from apoptosis [32, 35, 36]. However, the HDAC inhibitors SAHA and TSA were shown to regulate cell death by caspase-independent mechanisms [30, 37]. Apoptosis has also been linked to oxidative stress and DNA damage [35], mitochondrial disruption [35, 37], up-regulation and release of pro-apoptotic factors such as Bax and NOXA and Bim [25, 37, 38], down-regulation of anti-apoptotic factors [31, 37], and sensitisation to TRAIL-induced cell death [30]. Levels of the cytokines IL-6 and VEGF, secreted by the myeloma cells and the surrounding bone marrow stromal cells, are reduced by numerous HDAC inhibitors [25, 29, 30, 32, 39] which is in line with results from our experiments. Importantly, and in comparison to other HDAC inhibitors, CHR-3996 is potent, acting in the low nanomolar range. In addition it has oral bioavailability making it useful clinically and early Phase I trial data show it is well-tolerated [21].

Combination studies also support the use of HDAC inhibitors with other agents, with a number of studies showing promising anti-tumour activities [20, 38]. Targeting the proteotoxic stress is a potential treatment strategy in myeloma and combining bortezomib with inducers of protein misfolding promotes apoptosis in myeloma cells [40]. Previous laboratory investigations have demonstrated a synergistic relationship between various HDAC inhibitors – including tubacin [14], SAHA [41], and LBH589 [32] – with the proteasome inhibitor bortezomib. Like the majority of HDAC inhibitors these inhibitors either have broad spectrum activity or, as in the case of tubacin, activity specifically targeting HDAC6, and, therefore, inhibit the aggresome pathway [42]. Like bortezomib, they interfere with the cellular mechanisms for dealing with misfolded proteins, providing a rationale for their synergy [43]. The HDAC inhibitor described here, CHR-3996, has very low HDAC6 inhibitory activity therefore would not be expected to inhibit the aggresome pathway. In keeping with this, it was not found to be synergistic with bortezomib. A study of another HDAC inhibitor with a similar low HDAC6 inhibitory activity, R306465 [44], also described a lack of synergy between these agents.

Interestingly, in this study we have described a high degree of synergy both *in vitro* and *in vivo* between CHR-3996 and the aminopeptidase inhibitor tosedostat (CHR-2797). To further investigate the cellular basis of the synergy between these HDAC and aminopeptidase inhibitors we used expression profiling and found that the combination particularly affects level of expression of NFκB regulators. We have shown that while exposure to the HDAC inhibitor had minimal effects on p65 or p52 activation and nuclear translocation, the aminopeptidase inhibitor alone and in combination with the HDAC inhibitor strongly activates both canonical and non-canonical signalling pathways. NFκB is a key cancer driver and the activation of the NFκB pathway is generally thought to be associated with increased cell survival, opposing apoptosis in a wide range of cancers. Targeting the NFκB pathway is shown to be an effective strategy in both solid and haematological malignancies [45, 46]. However recent data counter this contention and indicate that, like our compounds, the anti-myeloma agent bortezomib and other proteasome inhibitors also activate NFκB signalling in myeloma cell lines [47]. Whether this increase in NFκB activity contributes to the apoptotic process or is a cytoprotective response triggered to protect the cell from programmed cell death has not been elucidated. NFκB activation is a self-limiting process mediated via a negative feedback mechanism; activation of this pathway up-regulates the expression of its own negative regulators. When the aminopeptidase and HDAC inhibitors are combined the activation of NFκB signalling is more rapid but is followed by increased expression of the NFκB regulators IκBα, A20, CYLD, and most markedly BIRC3. The strong induction of BIRC3 is associated with a decline in the levels of phosphorylated IκBα and active NFκB family members detectable in the nuclear compartment. Based upon these results we suggest that the presence of the HDAC inhibitor effectively turns off the cytoprotective NFκB response initially triggered by exposure to the aminopeptidase inhibitor, explaining why they are more effective when co-administered. Our data indicate that increased expression of NFκB regulators have an anti-myeloma effect, and this is supported by the high frequency of inactivating mutations found in these regulators (BIRC3, Traf2/3 and CYLD) in myeloma patients [48-51]. These mutations are associated with high levels of NIK activation and non-canonical NFκB signalling. Future work by gene expression profiling and testing the effect of these molecules in patient samples with such mutations would be useful to further confirm the mechanism of synergy.

In conclusion, the HDAC inhibitor CHR-3996 has been demonstrated to have potent anti-myeloma activity and is highly synergistic when combined with the aminopeptidase inhibitor tosedostat (CHR-2797), providing a good rationale for clinically combining these agents. Based on this work a phase I dose finding study of the two oral medications has been initiated. The combination of these compounds leads to rapid NFκB activation which is followed by induction of a negative feedback mechanism, the up-regulated expression of repressors of NFκB signalling, switching the cytoprotective response off. This suggests that the repressed expression of NFκB inhibitors like A20, CYLD, IκBα and BIRC3 is an important mechanism of myeloma cell survival, and confirms the central role of NFκB signalling in myeloma pathogenesis.

## MATERIALS AND METHODS

### Cell lines and reagents

Multiple myeloma cell lines, primary myeloma cells and bone marrow stromal cells were obtained and grown as previously described [23, 52]. Patient samples were obtained following informed consent. The aminopeptidase inhibitor tosedostat (CHR-2797), HDAC inhibitor CHR-3996 (Chroma Therapeutics Ltd, UK), and SAHA (Alexis Biochemicals, UK) were dissolved in DMSO to a stock concentration of 10 mM. Sodium Valproate (Sigma, UK) was dissolved in PBS at 2 M. Melphalan (Sigma, UK) was dissolved at 10 mM in 100% Ethanol adding concentrated hydrochloric acid drop-wise until completely dissolved. Bortezomib was prepared in DMSO at a concentration of 1 mM (Millennium Pharmaceuticals, USA). The caspase inhibitor Z-VAD-FMK (Calbiochem, UK) was supplied as a 10 mM solution and added to cells at a concentration of 50 μg/ml 1 hour prior to drug treatment.

### Cell proliferation, survival and cell cycle assays

Inhibition of proliferation was measured using a WST-1 assay (Roche, Germany) as per the manufacturer's instructions. Cell death was measured by flow cytometry using the AnnexinV:FITC Apoptosis Detection Kit I (BD Biosciences, UK) on a FACSCalibur^TM^. Cell cycle status was measured by fixing cells on ice in 70% ethanol, re-suspending in PBS and treated for 30 minutes at 37°C with 100 μg/ml RNase (Sigma, UK), then stained with 50 μg/ml Propidium Iodide (PI) and analysed by flow cytometry.

### Immunodetection

Protein was extracted from cells on ice in lysis buffer (1% sodium deoxycholate, 1% NP-40, 0.1% SDS, 50mM Tris, 150mM NaCl, 5mM EDTA, 30mM NaF, and 1mM PMSF supplemented with 1x protease inhibitor cocktail (Roche, Germany). The separation of cytoplasmic and nuclear fractions was performed using the Nuclear Extract Kit (Active Motif, Belgium). For histone extraction from cells the nuclear material was resuspended in lysis buffer with 0.2M Hydrochloric acid and left at 4°C overnight. Protein concentration was determined using a BCA protein assay (Pierce Biotechnology, USA). For immunoblotting 10μg (or 2 μg for acetyl-H3K9) was resolved by sodium dodecyl sulfate-polyacrylamide gel electrophoresis. This was transferred to PVDF membranes, blocked with 5% milk, and incubated with primary antibody: Caspase 3, 8, 9, (Cell Signaling, USA), acetyl-H3K9 (Upstate, USA), acetyl-Tubulin, actin (Sigma, UK), Puma, Ubiquitin, phosphorylated-IκBα (Ser32), IκBα, NFκB2, BIRC3 (Cell Signaling, USA), NOXA, EndoG (Calbiochem, USA), p65 (Santa Cruz, USA). Secondary antibodies used were anti-mouse or anti-rabbit conjugated to horseradish peroxidase (Amersham Biosciences, UK) and ECL-Plus (Amersham Biosciences, UK) used for detection. To measure NFκB binding activity within nuclear extracts the TransAM^TM^ NFkB kit (Active Motif, Belgium) was used according to the manufacturer's guidelines, adding 2 μg of nuclear extract per well.

### Cytokine measurement

Myeloma cell lines and bone marrow stromal cells were cultured separately or together for 24 hours then supernatant removed for cytokine analysis using Human VEGF and IL-6 Quantikine® ELISA kits (R&D Systems, USA) according to the manufacturer's guidelines.

### Proteasome assay

Cells were lysed on ice for 20 minutes in lysis buffer (20mM TrisHCl, 150mM NaCl, 1mM EDTA, 1mM EGTA, 1% Triton, supplemented with 1x protease inhibitor cocktail (Roche, Germany). The protein concentration was determined by BCA assay (Pierce, UK) and 25 μg of protein was mixed with substrate for the chymotryptic activity of the proteasome (Suc-Leu-Leu-Val-Tyr-AMC at 75 μM). The fluorescence (using 355nM excitation and 460nM emission wavelengths) was measured on a Mithras LB940 plate reader (Berthold Technologies, Germany). Data from each well was collected over a 1 second period, repeating the measurement every 120 seconds for a total of 48 measurements performed at 37°C.

### Gene expression studies

RNA was extracted from cells with the RNeasy Plus Mini kit (Qiagen, UK). RNA quality and quantity was determined using a 2100 Bio-analyser (Agilent, USA). For microarray experiments 100 ng total RNA was amplified using a 2-cycle target biotin labelling kit (Affymetrix, USA) and cRNA was hybridized to Human Genome U133 Plus 2.0 expression arrays. The arrays were washed on an Affymetrix Fluidics Station 450 and scanned with a Gene Chip Scanner 3000. Normalization and data analysis was performed using dChip software (http://www.dchip.org/). Supervised analysis was performed using dChip to determine genes differentially expressed in the control (C) or treated (T) cells. Comparison criteria used were: C/T fold difference (greater or lower) is equal or greater than 2x mean difference T-C or C-T is greater than 100, p value is less than 0.05.

### Quantitative PCR

cDNA was synthesised from 500 ng RNA using the High Capacity cDNA Reverse Transcription kit (Applied Biosystems, UK). Primer sequences were designed over exon/exon boundaries for: β-actin F5′ ccctggcacccagcac R5′ gccgatccacacggagtac

*p21* F5′ CTGGAGACTCTCAGGGTCGAA R5′ GCGTTTGGAGTGGTAGAAATCTG. Thermal cycling conditions were 10 minutes at 95°C, 40 cycles at 95°C for 15 seconds followed by 1 minute at 60°C on a 7500 Fast Real-Time PCR System using Power SYBR® Green PCR mastermix. All reagents, software, and equipment were supplied by Applied Biosystems, UK, and used according to the manufacturer's guidelines.

### Xenograft murine model

A breeding colony of NOD/SCID IL2R gamma^null^ (obtained from The Jackson Laboratory, USA) were housed and monitored at the Biomedical Science Unit at the Institute of Cancer Research. All experimental procedures and protocols have been approved by local ethical review at the Institute of Cancer Research and the Home Office. The mice were inoculated subcutaneously in the right flank with 2×10^6^ H929 myeloma cells in 50 μL RPMI-1640 and 50 μL Matrigel^TM^ Basement Membrane Matrix Growth Factor Reduced (Becton Dickinson). The mice were assigned into the following four treatment groups (10 animals per group): no treatment, tosedostat 75 mg/kg, CHR-3996 30 mg/kg, and tosedostat 75 mg/kg with concomitant CHR-3996 30 mg/kg. Both compounds were administered daily beginning four days after the tumour cells were inoculated; tosedostat by intra-peritoneal injection and CHR-3996 per oral. Caliper measurements of the longest perpendicular tumour diameters (length) and width were performed every other day to estimate the tumour volume using the following formula representing the 3D volume of an ellipse: 1/2 × (length) × (width)^2^.

## SUPPLEMENTARY TABLES


