# Supplementary material for: The combination of HDAC and aminopeptidase inhibitors is highly synergistic in myeloma and leads to disruption of the NFκB signalling pathway
Source: Oncotarget. 2013 Aug 12;6(19):17314–27. doi: 10.18632/oncotarget.1168 (PMC4627310; doi:10.18632/oncotarget.1168)
Supplement: Supplementary file 1 [file oncotarget-06-17314-s001.pdf]

The combination of HDAC and aminopeptidase inhibitors is highly synergistic in Myeloma and leads to disruption of the NFκB signalling pathway – Smith et al

Supplementary Table 1: The LC_{50} doses for myeloma cell lines treated with HDAC inhibitors.

Cell line	CHR-3996 LC50 (nM)	SAHA LC50 (μM)	Na Valproate LC50 (mM)
H929	97.6	0.51	1.38
KMS11	30.3	0.17	0.82
LP-1	87.0	0.51	3.14
MM1s	43.7	1.84	10.0
RPMI-8226	33.0	0.67	1.37

Supplementary Table 2: GEP changes following HDAC inhibition in myeloma cells.

H929 were treated with CHR-3996 (250 nM) for 24 hours then analysed on a U133 Plus 2.0 Array (Affymetrix) and a supervised analysis performed with dCHIP comparing cells treated with the HDAC inhibitor to untreated cells. All p values were less than 0.05.

**Probe ID**	**Gene**	**Fold change**	**Function**
Cell cycle			
236313_at	CDKN2B (p15)	18.71	Inhibits cyclin dependent kinases
202769_at	Cyclin G2	18.65	Negatively regulates cell cycle
219534_x_at	CDKN1C (p57)	15.39	Inhibits cyclin dependent kinases
222494_at	FOXN	2.34	Cell cycle arrest
202284_s_at	CDKN1A (p21)	1.51	Cell cycle arrest
201853_s_at	CDC25B	-2.14	Entry into mitosis
202870_s_at	CDC20	-2.17	Cell cycle control
204826_at	cyclin F	-2.25	Cell cycle progression
205034_at	cyclin E2	-2.55	Cell cycle progression
226396_at	CDK3	-2.71	S phase entry
204244_s_at	DBF4/ASK	-2.86	Cell cycle progression
204092_s_at	Aurora kinase A	-3.07	Cell cycle progression
214710_s_at	cyclin B1	-3.15	Cell cycle progression
201202_at	proliferating cell nuclear antigen	-3.58	Cell cycle progression
208712_at	cyclin D1	-3.92	Cell cycle progression
203214_x_at	CDK1	-3.98	Cell cycle progression
205393_s_at	CHK1	-6.51	Cell cycle checkpoint
204318_s_at	GTSE1	-5.17	Cell cycle regulation, negatively regulates p53
209464_at	Aurora kinase B	-5.31	Cell cycle progression
203968_s_at	CDC6	-6.9	Initiation of DNA replication
203418_at	cyclin A2	-8.7	Cell cycle progression
NFκB			
210538_s_at	BIRC3	23.51	Negative regulator of NFκB signalling
223218_s_at	IκB zeta	7.49	Inhibits NFκB transcriptional activity
221903_s_at	CYLD	2.47	Negative regulator of NFκB signalling
201391_at	TRAF1	-2.14	TNF-induced NFκB activation
206641_at	BCMA	-3.1	BAFF induced NFκB activation
p53			
225912_at	TP53 inducible nuclear protein 1	20.91	Mediates p53 cell cycle arrest and apoptosis
202672_s_at	ATF3	14.69	Cell cycle arrest in response to DNA damage
218346_s_at	Sestrin 1	3.36	Activates mTOR inhibitors
223196_s_at	Sestrin 2	3.23	Activates mTOR inhibitors
211692_s_at	PUMA	3.12	p53 mediated apoptosis
228006_at	PTEN	2.93	Antagonises PI3K/Akt signalling and MDM2-led p53 degradation
203132_at	RB1	2.09	Negative regulator of cell cycle
33322_i_at	14-3-3 sigma	2	p53 regulated cell cycle arrest
218403_at	P53CSV	-2.16	Protects cells from DNA damage-mediated apoptosis, up-regulated in a high percentage of myeloma patients
1552518_s_at	MDM2	-3.7	Promotes p53 degradation

208712_at	cyclin D1	-3.92	Cell cycle progression
Stress			
236990_at	PERK	15.25	Mediates ER stress response, activates EIF2alpha
209383_at	DDIT3 (CHOP)	13.3	Stress-induced apoptosis
239045_at	IRE-1	5.29	Mediates the ER stress response
220761_s_at	JIK	3.41	Binding partner of IRE-1
226941_at	ATF6	2.77	UPR transcription factor
230031_at	BiP	2.47	ER protein folding and assembly
212984_at	ATF2	2.17	Anti-proliferative/apoptotic role in response to stress stimuli
Apoptosis			
1552703_s_at	caspase 1	22.99	Caspase-mediated apoptosis
225606_at	BIM	8.3	Binds and antagonises Bcl-2 family members
212593_s_at	programmed cell death 4	8.06	Pro-apoptotic and tumour suppressor
204859_s_at	APAF1	4.22	Central to the apoptosome, activates Caspase9
204131_s_at	FOXO3	3.3	Triggers apoptosis by increasing expression of pro-apoptotic genes
211692_s_at	PUMA	3.12	Binds and antagonises Bcl-2 family members
203984_s_at	caspase 9	2.37	Caspase-mediated apoptosis
202095_s_at	BIRC5 (survivin)	-4.39	Inhibits apoptosis
Wnt			
238624_at	Nemo like kinase	9.14	Antagonises Wnt signalling
204602_at	DKK1	7.6	Antagonises Wnt signalling
200765_x_at	Alpha-catenin	5.01	Regulates the transcriptional activity of beta-catenin
201908_at	Dishevelled 3	2.31	Antagonises Wnt signalling
226191_at	GSK3 beta	2.11	Antagonises Wnt signalling
Autophagy			
208786_s_at	LC3B	5.3	Autophagosome formation
221478_at	BNIP3L	4.22	Initiates autophagy by disrupting Bcl-2-Beclin1 complexes
223677_at	APG10 autophagy 10-like	3.29	Autophagosome formation
202512_s_at	APG5 autophagy 5-like	2.22	Autophagosome formation
Histone modifying			
205659_at	HDAC9	8.26	Histone deacetylase
222777_s_at	WHSC1 (MMSET)	4.69	Histone methyltransferase
241458_at	Huntingtin interacting protein B	3.44	Histone methyltransferase
202455_at	HDAC5	2.82	Histone deacetylase
213320_at	PRMT8	-2.1	Histone methyltransferase
202182_at	GCN5	-2.35	Histone acetyltransferase
204027_s_at	methyltransferase like 1	-3.29	Histone methyltransferase
Various			
205698_s_at	MEK6	5.07	Activates p38 (cell cycle arrest and apoptosis)
203836_s_at	ASK1	2.26	Activates p38 (cell cycle arrest and apoptosis)
212271_at	p38	2.08	Cell cycle arrest and apoptosis
207121_s_at	ERK3	-2.1	Proliferation in response to external growth factors
205945_at	IL6R	-2.7	Response to IL-6

204379_s_at	FGFR3	-4.28	Proliferation in response to external growth factors

Supplementary Table 3: CHR-3996 and HDAC inhibitors SAHA and Sodium Valproate (VA) are highly synergistic with aminopeptidase inhibitor CHR-2797.

H929 and RPMI-8226 cells were treated with a range of concentrations of HDAC inhibitor (**HDACi**, CHR-3996, SAHA or Sodium Valproate (VA)) and aminopeptidase inhibitor (**APi**) tosedostat (CHR-2797) over 96 hours after which the proliferation was measured by WST-1 assay. The Combination Index (CI) was calculated by a full 16 point Chou-Talalay analysis and the value for the 50% fraction given. When added in combination the drugs were either added concomitantly or 24 hours prior to one another. A CI of <0.8 is defined as synergistic, 0.8-1.2 additive, and >1.2 antagonistic.

**HDACi**	**Cell line**	**HDACi + APi: concomitant**	**HDACi + APi: APi 24 hr prior**	**HDACi + APi: HDACi 24 hr prior**
CHR3996	H929	0.53	0.38	0.83
	RPMI-8226	0.36	0.19	0.79
SAHA	H929	0.68	0.24	0.66
	RPMI-8226	0.33	0.15	1.44
VA	H929	0.29	0.17	0.33
	RPMI-8226	0.14	0.15	0.78
